# PdMATE16 and PdMATE35 are essential for the accumulation of multiple flavonoids in tree peonies

**DOI:** 10.3389/fpls.2025.1703357

**Published:** 2025-11-19

**Authors:** Meng Yuan, Xinrui Wei, Fucheng Peng, Qun Wang, Lin Zhou, Yan Wang

**Affiliations:** Key Laboratory of Tree Breeding and Cultivation of National Forestry and Grassland Administration, Research Institute of Forestry, Chinese Academy of Forestry, Beijing, China

**Keywords:** mate, gene family, transport, flavonoid, *Paeonia delavayi* var. *lutea*, flower color

## Abstract

As a plant with both medicinal and edible uses, peonies are not only valued for their ornamental qualities, but also for the high economic value of the flavonoids found in their petals. However, the regulatory mechanisms governing flower color and flavonoid transport remain unclear. The multidrug and toxic compound extrusion (MATE) family of proteins are important transport protein involved in the transport of a variety of secondary metabolites in plants. Based on transcriptomic data, this study identified 37 MATE genes from the transcriptomic data of *Paeonia delavayi* var. *lutea*. Molecular docking and site-directed mutagenesis results predict that PdMATE16 and PdMATE35 can bind multiple pigments. Overexpression and virus-induced gene silencing (VIGS) experiments further confirmed that PdMATE16 and PdMATE35 could transport a variety of flavonoid compounds, including flavone glycosides, flavonol glycosides, chalcone glycosides and anthocyanin glycosides. These substances not only give peonies their yellow color, but are also the main active ingredients in medicinal applications. In addition, several transcription factors that may be involved in regulating MATE gene expression were screened. Our research confirms the key role of PdMATE16 and PdMATE35 in flavonoid transport in peonies, clarifies the regulatory mechanism underlying the transport processes involved in peony coloration, and may thus contribute to the improvement of ornamental and medicinal plants.

## Introduction

Flower color is one of the important ornamental traits of ornamental plants and has great economic value. The secondary metabolites involved in the formation of flower color include flavonoids, anthocyanins, carotenoids etc. The content and proportion of these substances in petals directly affect the color phenotype of plants. In addition, these secondary metabolites also have pharmacological activity and are often used as natural extracts in food, medicine, or cosmetics (Kopustinskiene et al., 2020; Li et al., 2009; Zhao et al., 2023). Therefore, studying the regulatory mechanisms of flavonoid transport may provide meaningful guidance for the ornamental and medicinal value of peonies. Currently, most of the studies on flower color focus on the components and their synthetic pathways, with relatively few studies targeting the downstream transporter processes. Currently, the recognized transporter proteins in plants include ATP-binding cassette (ABC) transporter proteins, Na+ or H+ coupled transporter proteins of the major facilitator superfamily (MFS), small multidrug resistance (SMR) transporter proteins, and multidrug and toxic compound extrusion (MATE) transporter proteins (Kuroda and Tsuchiya, 2009; Remy and Duque, 2014).

MATE transporter proteins are widely found in a variety of organisms and are important membrane proteins. MATEs are mainly responsible for the transport of a wide range of compounds inside and outside the cell, including drugs, toxins, and secondary metabolites, and help to protect the cell from toxic compounds (Upadhyay et al., 2019). Their transport mechanism is usually driven by a proton gradient, i.e., they utilize the difference in proton concentration on both sides of the cell membrane to provide energy to drive the transport of compounds. The first plant MATE transporter protein, AtALF5, was isolated in Arabidopsis (*Arabidopsis thaliana*) in 2001 (Diener et al., 2001). Currently, members of the MATE family have been identified in a variety of plants, such as Arabidopsis, tobacco (*Nicotiana tabacum*), wheat (*Triticum aestivum*), potato (*Solanum tuberosum*), citrus (*Citrus clementina*), pear (*Pyrus pyrifolia*), radish (*Raphanus sativus*), soybean (*Glycine max*) and *Torreya grandis* (Gani et al., 2021; Sheng et al., 2023; Huang et al., 2021; Liu et al., 2022; Gao et al., 2024; Shen et al., 2024; M’mbone et al., 2018).

Several MATE proteins have been shown to transport secondary metabolites in plants. In soybean, GmMATE100 is responsible for the transport of soyasaponins from the cytoplasm to the vesicles. NtMATE1 can efflux nicotine through a proton gradient (Shoji et al., 2009). AtDTX41/TT12 in Arabidopsis controls the transport of anthocyanin cyanidin-3-*O*-glucoside in the seed coat (Marinova et al., 2007; Debeaujon et al., 2001). MtMATE1 and MtMATE2 are involved in flavonol transport in tobacco and influence plant growth and development (Zhao et al., 2011). VvMATE in grape (*Vitis vinifera*), FaTT12 in strawberry (*Fragaria* x *ananassa*), and MdMATE1 and MdMATE2 in apple (*Malus* x *domestica*) can be involved in proanthocyanidins transport (Pérez-Díaz et al., 2014; Chen et al., 2018; Frank et al., 2011). In addition, different MATE transporter proteins differ in their specificity for compounds. For example, MATE transporter proteins in persimmon preferentially transport tannin precursors, which affect the astringent flavor of the plant (Liu et al., 2024, 2023). In summary, the current studies are mostly focused on model plants, and fewer involve ornamental plants, especially in peonies, where relevant studies have not yet been conducted.

**Figure 1:**
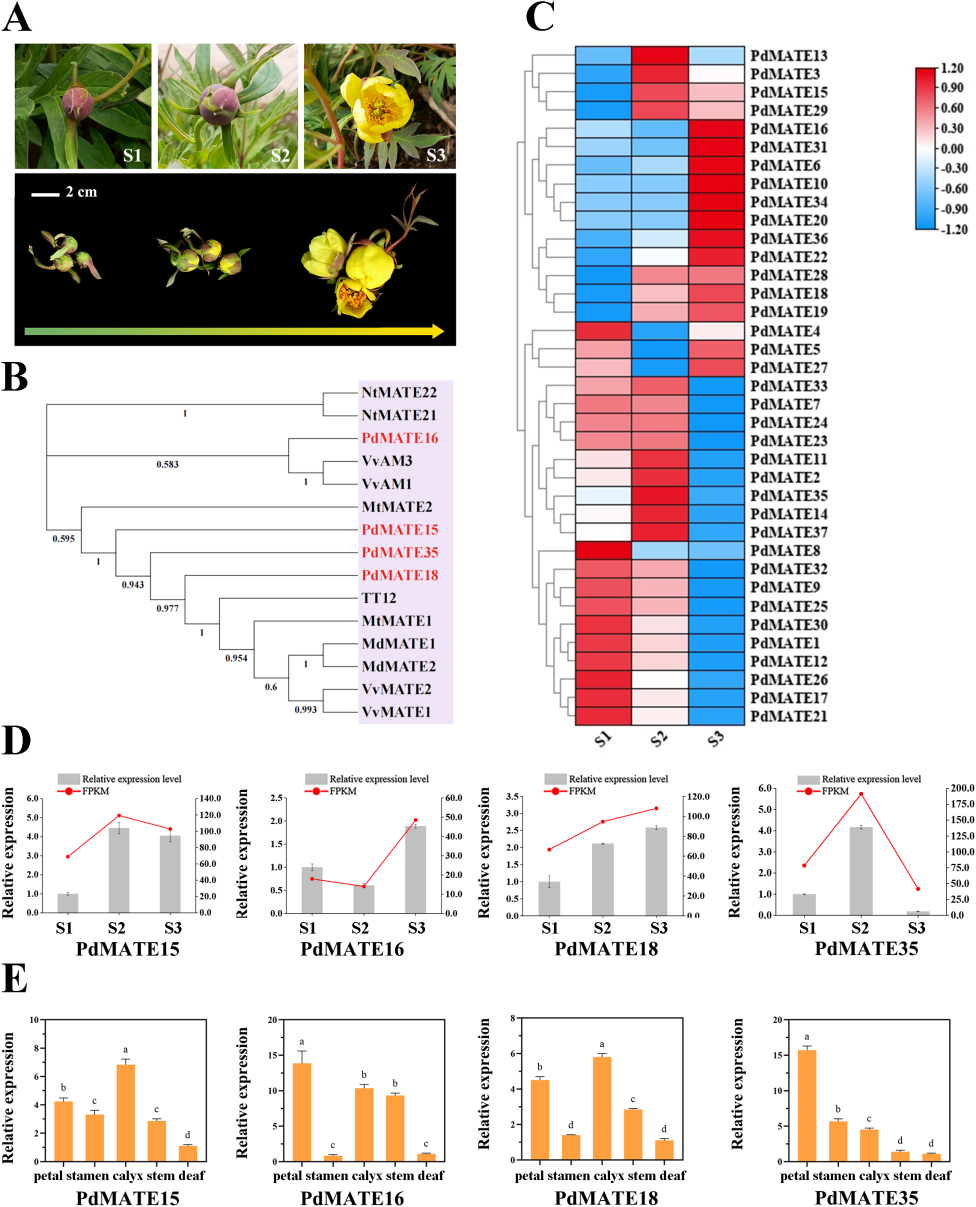
Identification and screening of transporter flavonoid MATE in *P. delavayi* var. *lutea*. **(A)** Petal phenotypes of *P. delavayi* var. *lutea* at three flowering stages. S1: Immature tight bud; S2: mature flower at the closed-bud stage; S3: fully opened flower. Bar = 2 cm. **(B)** Phylogenetic tree was constructed using the neighbor-joining method of PdMATE with identified flavonoid-related MATEs from other plants. **(C)** Expression pattern analysis of PdMATE gene family members in different flower developmental processes. **(D)** The expression profiles of five PdMATE genes in different developmental stages of *P. delavayi* var. *lutea* were analyzed using qRT-PCR. **(E)** The expression profiles of five PdMATE genes in different *P. delavayi* var. *lutea* tissues were analyzed by qRT-PCR. An asterisk indicates a significant difference. Different letters indicate significant differences (*P* < 0.05).

*Paeonia delavayi* var. *lutea* is the most widely distributed and colorful wild species in the peony group (**Figure 1A**). It is an irreplaceable genetic resource in the cultivation of ornamental peonies, as it is the source of most of the yellow and purple peony varieties available in the market nowadays. The root of *P. delavayi* has a variety of medicinal components, and it is one of the original plants of the traditional Chinese medicine “Danpi” (Moutan Cortex), which has a high medicinal value. It has been proved that the main chromogenic substances in *P. delavayi* var. *lutea* are flavones, flavonols and chalcones (Yang et al., 2020). The current studies focused on the composition and synthesis pathways of these chromogenic components, but the study on the regulation mechanism of petal pigment transport mediated by the MATE gene has not been reported yet. The systematic identification and analysis of the MATE family and their roles in the petal coloration of *P. delavayi* var. *lutea* remains to be explored.

In this study, we identified a total of 37 MATE genes by transcriptome data and comprehensively analyzed their gene structures and conserved motifs. Based on phylogenetic relationships and molecular docking, we initially predicted two MATE proteins, PdMATE16 and PdMATE35, that might be involved in the transport of floral pigments in the petals of *P. delavayi* var. *lutea.* Codon preference analysis confirmed that tobacco was the best receptor for functional validation of *P. delavayi* var. *lutea*. Through overexpression experiments in tobacco and VIGS silencing experiments in peony, we confirmed that PdMATE16 and PdMATE35 could transport a variety of flavonoid compounds, including flavones, flavonols, chalcones and anthocyanins. These substances not only give peonies their yellow color but are also the main active ingredients in medicinal applications. This suggests that PdMATE16 and PdMATE35 genes play an important role in petal pigment transport in *P. delavayi* var. *lutea*. In addition, several transcription factors that might be involved in the transporter process, such as MYB, BHLH, and WRKY, were screened out by weighted gene co-expression network analysis, but their specific regulatory mechanisms need to be further investigated. In summary, this study provides new insights into the function of the MATE gene in *P. delavayi* var. *lutea* and provides new clues for further enhancing the ornamental and medicinal value of yellow peonies.

## Materials and methods

### Plant materials and sampling

The *P. delavayi* var. *lutea* petal material utilized in the experiment was obtained from Shangri-La County, Yunnan, China (N: 27°36′16.56″; E: 99°46′16.90″; Altitude: 3300 m). The sampling stage was divided into three stages according to the growth status: immature tight buds (S1), mature blooms at the closed-bud stage (S2), and fully opened flowers (S3) (**Figure 1A**). Samples were stored in liquid nitrogen immediately after harvesting and subsequently transported on dry ice and placed in a -80 °C refrigerator for all subsequent tests.

Peony cultivars ‘High Noon’ and ‘Xue Ying Tao Hua’ used in the gene silencing experiment were planted in Xishan National Forest Park, Beijing, China (N: 39°58′18.17″; E: 116°11′51.20″).

### MATE gene family identification and sequence analysis

The MATE sequence of the model plant Arabidopsis (**Supplementary Table S6**) was obtained from the TAIR database (https://www.arabidopsis.org/) as a query sequence, and a BLASTP search was conducted in the *P. delavavi* var. *lutea* transcriptome (Number: PRJNA1240698). Sequences with missing structural domains, as well as those with identity and coverage regions less than 50%, were removed from the search results. The final 37 MATE gene models that remained were utilized for further analysis. The PdMATE protein subcellular localization using pSORT (https://www.genscript.com/psort.html) and Cell PLoc (http://www.csbio.sjtu.edu.cn/bioinf/Cell-PLoc-2/) was employed. Multiple sequence comparison in ClustalW was used to estimate the sequence similarity of PdMATE proteins within and between classes.

The identification of conserved motifs predicted by MEME (http://meme-suite.org/-tools/meme)-55 was visualized using TBtools with MEME parameters set as follows: maximum number of motifs: 10, optimal motif width: <50; other parameters were kept at default values. The transmembrane structural domains of proteins encoded by PdMATE family genes were analyzed using SignalP 4.1 (https://services.healthtech.dtu.dk/services/SignalP-4.1/) and TMHMM2.0 Server (https://services.healthtech.dtu.dk/services/TMHMM-2.0/).

### Phylogenetic analysis

The Arabidopsis MATE gene family sequences, as well as other MATE genes in the literature that have been validated to have a florochrome transporter function, were used to construct an evolutionary tree with the PdMATE genes in order to screen for PdMATE genes that potentially have a flavonoid transporter function. All MATE amino acid sequences were compared using ClustalW (**Supplementary Table S6**). The evolutionary tree was constructed by MEGA-X (https://www.megasoftware.net/) with the model set to p-distance, and the construction method was the maximum likelihood (ML) method using 1000 bootstrap replicates to assess the topology and reliability of the tree.

### Molecular docking

Small molecule ligand models for molecular docking were downloaded from PubChem (https://pubchem.ncbi.nlm.nih.gov/). PdMATE16 and PdMATE35 were modelled ed using SwissModel. Molecular docking was performed using Autodock software. The smaller values of the results obtained demonstrate the higher binding strength of the ligand (**Supplementary Table S2**). The type of interaction force for the molecular docking results was predicted using PLIP (https://plip-tool.biotec.tu-dresden.de/plip-web/plip/index). Finally, visualization was performed using PyMOL.

### Codon preference analysis

In this study, CodonW software was used to calculate the codon bias indexes: codon adaptation index (CAI), effective codon count (ENc), synonymous codon position 3 G and C content (GC3s), relative usage (RSCU), and G/C content (GC). The selective differences in codon usage.

between *P. delavayi* var. *lutea* and the model crops were obtained by calculating the ratio of codon usage frequency. A ratio of 0.5-2.0 indicates a small difference, and vice versa, a large difference.

### Isolation of RNA, synthesis of first-strand cDNA, and gene expression analysis

All primers in this experiment were designed by Oligo 7 software (**Supplementary Table S7**). Total RNA extraction was performed using a kit from Huayueyang Biotechnology (Huayueyang Biotechnology, Beijing, China). Reverse transcription was performed using the One-step gDNA Removal and cDNA Synthesis SuperMix for qPCR (TransGen Biotech, Beijing, China) kit. qRT-PCR internal reference was the PP2A gene, and reagents were used with the SYBR Premix Ex Taq TM II kit (TaKaRa, China), and the whole experiment was completed on a LightCycler 480 platform. Three biological replicates and three technical replicates were performed for each reaction. Relative gene expression levels were calculated by comparing the threshold cycling (Ct) values.

### Stable transformation of tobacco

The complete CDS sequences of *PdMATE16* and *PdMATE35* were constructed into the plant expression vector pCAMBIA1302 and transformed into GV3101. The correctly sequenced strains were selected and transformed into tobacco using the leaf-disk method. The histocultured seedlings were allowed to root and then transplanted to an incubator for culture until flowering. Subsequent experiments were carried out using PCR-detected positive plants.

### Virus-induced gene silencing of PdMATEs in peony

The pTRV2-GFP vectors for *PdMATE16* and *PdMATE35* were constructed using a seamless cloning approach. The correctly sequenced candidate MATE genes were transferred into GV3101 and cultured using YEP medium with antibiotics until the liquid OD600 = 1.6. Centrifugation and addition of resuspension (10 mM MES, 100 µM AS, 10 mM MgCl_2_) adjusted the OD600 to 1.0. The resuspension of the candidate genes was mixed in an equal volume with the pTRV1 bacterial broth and incubated in the dark for 3 h and then injected into peony buds. The injection volume was 3 ml per flower, and the peony buds were shaded for 24 h. Phenotypic observation, photographs and subsequent experiments were performed after flowering.

Genes with a fold change ≥ 2 and a false discovery rate (FDR) < 0.05 were screened and identified as significant differentially expressed genes (DEGs). Based on the expression data of these DEGs in the transcriptome, a heatmap of the flavonoid metabolic pathway was constructed (**Supplementary Figure S1**). Among these DEGs, transcripts with high correlation (Pearson correlation coefficient > 0.75) with major pigment substances and high expression levels in the transcriptome were selected as genes for quantitative detection (**Supplementary Table S8**). For details on flavonoid content in petals of *P. delavayi* var. *lutea* at different flowering stages, see **Supplementary Table S9**.

### Determination of flavonoid components and total anthocyanin contents

The total flavonoid content in this study was determined using the BC1335 kit from Solebo. 0.1g of sample was taken and pulverized, and added to 1ml of extraction solution and extracted using ultrasonication. The sonication time was set at 30 minutes, power 300W and temperature 60 °C. The extract was centrifuged, and the volume of the extract was set at 0.5 ml. After centrifugation, the extract was used to finalize the volume to 1 ml. 200µ of the supernatant was taken and used to determine the absorbance value at A470. A standard curve was established by dilution using rutin as a standard and the calculation of the flavonoid content of the samples was carried out based on the standard curve. In addition, the flavonoid metabolome was assayed by MetWare (http://www.metware.cn/) based on a UPLC-MS/MS assay platform and a self-built database.

The total anthocyanin content was calculated according to the formula Q = Vx(A530-0.25xA657)/M, where V is the volume of solution (mL) and M is the fresh weight of the sample (g). The specific experimental method was as follows: a fresh sample of 0.1 g of plant petals was ground and extracted using 2 ml of acidified methanol with the addition of 1% HCL protected from light, for 18 h. After centrifugation, 1 ml of the supernatant was taken for the determination of absorbance.

### Data analysis

Statistical analysis was performed using SPSS (SPSS Inc., Chicago, IL, USA) and GraphPad PRISM software (GraphPad Software Inc., La Jolla, CA, USA) to assess the statistical significance of the differences between the mean values. Differences with a *p*-value less than 0.05 were considered significant.

## Results

### Identification and phylogenetic analysis of PdMATE

Screening gene families using genomes of closely related species may lead to inaccurate annotation (Wu et al., 2024; Nevers et al., 2025). Currently, the publicly available high-quality peony genomes are those of *P. suffruticosa* ‘Fengdan’ and *P. suffruticosa* ‘Luoshen Xiaochun’ (Lv et al., 2020; Yuan et al., 2022), which exhibit significant genetic differences from *P. delavayi* var. *lutea*. Therefore, we chose to use transcriptome data for screening. This approach not only directly reflects gene expression activity but also improves the accuracy of functional gene selection (Dong et al., 2024; Guo et al., 2025).

**Figure 2:**
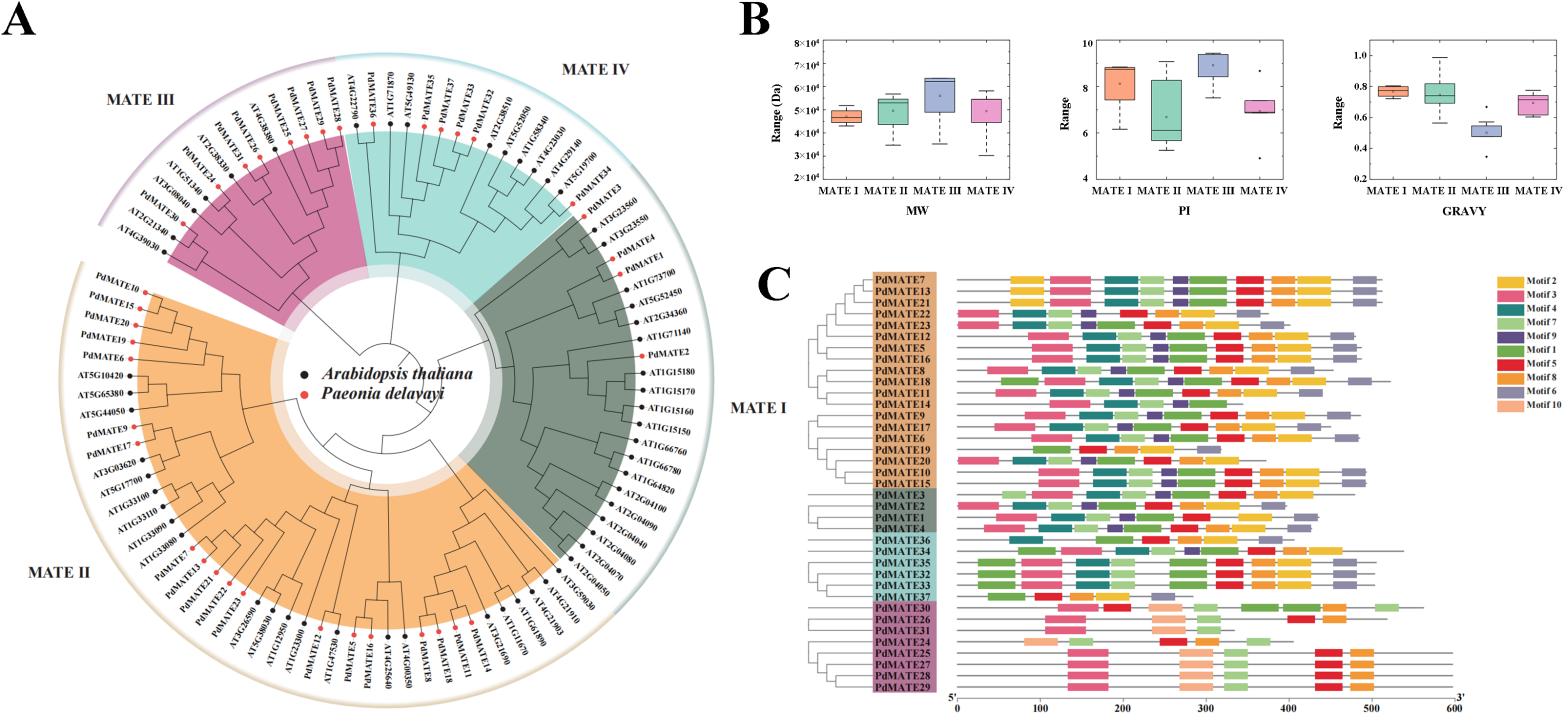
Identification and phylogenetic analysis of PdMATE. **(A)** Phylogenetic analysis of PdMATE proteins. **(B)** Visualization of physicochemical properties of members of PdMATE on a subfamily basis. **(C)** Schematic representation of the phylogenetic relationships and conserved motifs analysis in PdMATE proteins.

We identified a total of 37 MATE transporter proteins from the transcriptome of *P. delavayi* var. *lutea* and named them PdMATE1-PdMATE37 according to their transcriptome IDs, respectively. The length of the PdMATE proteins ranged from 197 to 642 amino acids (**Supplementary Table S1**). A phylogenetic tree was constructed by MEGA of the 37 PdMATEs with the Arabidopsis MATE gene family, and the results showed that the PdMATE family was classified into four subfamilies, with the MATE II subfamily having the largest number of members (**Figure 2A**; **Supplementary Table S1**). The total hydrophilicity coefficients of the PdMATE proteins were all greater than 0, which indicated that all PdMATE proteins were hydrophobic (**Figure 2B**). Therefore, all proteins of the MATE family may exercise their physiological functions as membrane proteins. A total of 10 distinct motifs were identified in the 37 MATE genes (**Figure 2C**). The distribution of conserved elements among family members within each subfamily was similar, but there were large differences between subfamilies, indicating a high degree of structural conservation among homologous genes. In some subfamilies, there were members with greater than 80% similarity to each other, such as the MATE II and MATE IV subfamilies of MATE, suggesting that the gene duplication rate in these subfamilies was high (**Supplementary Figure S2**).

Transmembrane region analysis of PdMATE family proteins using TMHMM 2.0 Server showed that all members of the PdMATE family proteins are transmembrane proteins, and most of the PdMATE proteins have more than 10 transmembrane regions (**Supplementary Figure S3**). Subcellular localization analysis of PdMATE family proteins using PSORT showed that, among them, PdMATE14 and PdMATE26 might be distributed on chloroplasts, PdMATE20 might be distributed on vesicle membranes, and the rest of the members were distributed on plasma membranes (**Supplementary Table S1**).

### Screening and expression pattern analysis of flavonoid-related PdMATE

By constructing an evolutionary tree with MATE transporter proteins with verified functions in other plants, four PdMATE members were screened: *PdMATE15, PdMATE16, PdMATE18*, and *PdMATE35*, which may have the function of transporter pigments (**Figure 1B**). Combined with the transcriptome data measured by the group in the previous period (number: PRJNA1240698), the heat map of MATE family gene expression in petals of *P. delavayi* var. *lutea* during three flowering periods was produced, and the results showed that, in the second branch, the genes showed significant high expression in the late stage of flower development, and most of them peaked at the S3 period (**Figure 1C**). Among them, the expression abundance of *PdMATE16* and *PdMATE18* was 2.7-fold and 1.6-fold higher than that of S1 during the S3 period, respectively. In the fourth branch, the genes were significantly highly expressed in the S1 and S2 periods, but the expression levels of the genes showed a decreasing trend with time, and most of the genes peaked in the S2 period. Among them, the expression abundance of *PdMATE35* in S2 was 2.4-fold and 4.6-fold that of S1 and S3, respectively.

QRT-PCR assays using cDNA from petal parts of *P. delavayi* var. *Lutea* at different flowering stages showed that the quantitative results of four candidate MATE transport genes matched the trend of the transcriptome data (**Figure 1D**). Subsequently, the expressions of the candidate MATE transporter genes were determined in different tissues of *P. delavayi* var. *lutea* (**Figure 1E**). The results showed that *MATE16* and *MATE35* had the highest expression in petals and showed high tissue-specific expression, and it was hypothesized that they might be involved in the pigment transport process in petals of *P. delavayi* var. *lutea*.

### Co-expression network analysis and correlation analysis of candidate MATE genes

**Figure 3:**
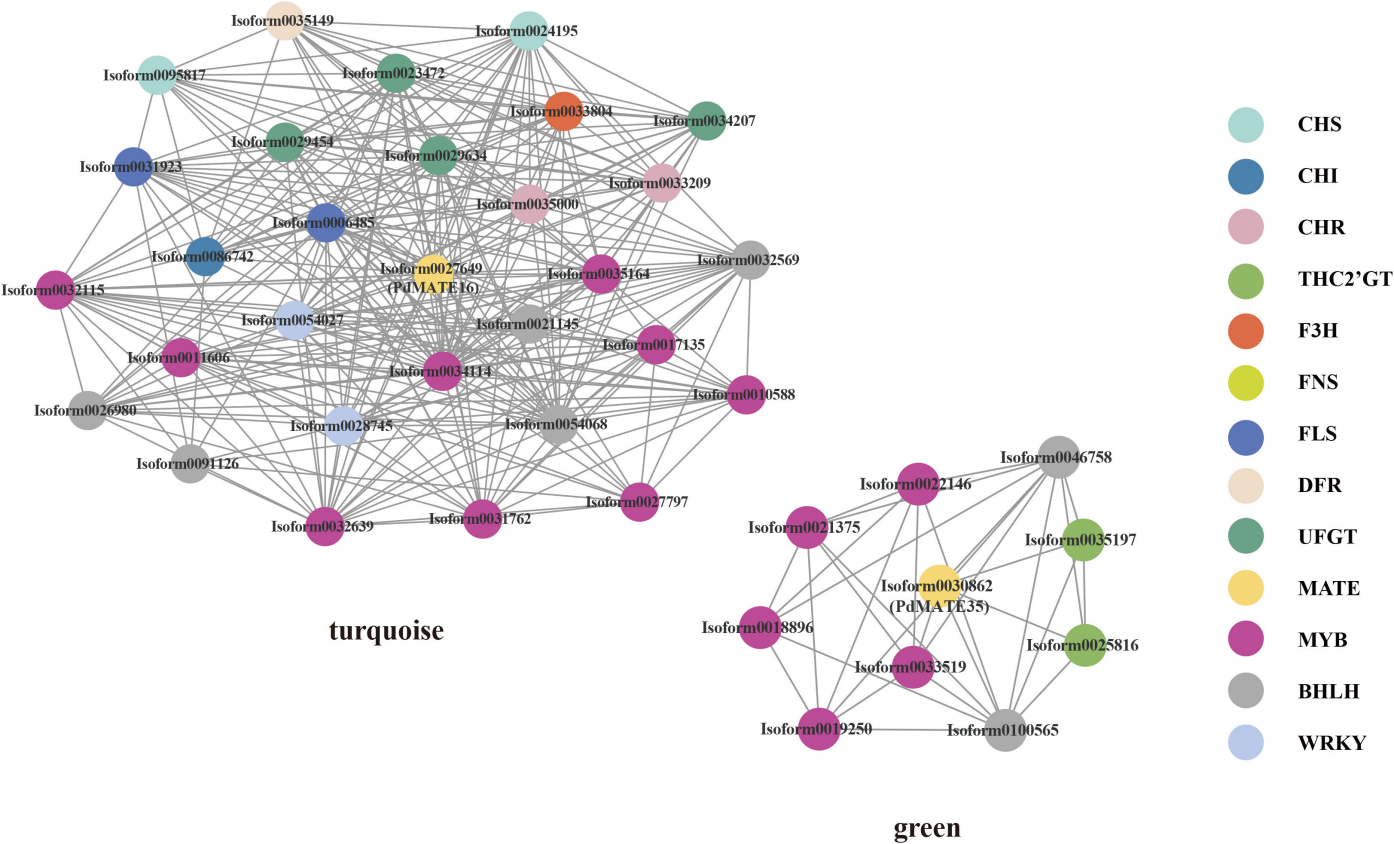
Co-expression network represents the relationships between PdMATEs and possible transcription factors predicted by WGCNA methods. Different colors represent different genes.

In order to further confirm the potential function of the candidate PdMATE gene in the transport of flavonoids, a total of 23 expression modules of information were obtained from *P. delavayi* var. *lutea* flower developmental process based on WGCNA analysis. Among them, *PdMATE16* was located in the turquoise module and *PdMATE35* in the green module (**Figure 3**). The co-expression network diagram showed that *PdMATE35* was co-expressed with THC2’GT, and *PdMATE16* was co-expressed with several structural genes in the flavonoid synthesis pathway, such as *CHS, CHI, F3H, FLS, DFR, UFGT*, etc., during flower development. The results also further confirmed that the candidate transporter genes might be involved in the transport and accumulation of flavonoids in *P. delavayi* var. *lutea*.

In order to better elucidate the relationship between the candidate transporter genes and the key color-developing substances in *P. delavayi* var. *lutea*, the transcriptome and metabolome were co-analyzed (**Supplementary Figure S5**). Co-expression of two candidate MATE transporter genes with key pigments in *P. delavayi* var. *lutea* revealed strong correlations with a total of 46 substances. We focused on flavones, flavonols, and chalcones among them. It can be found that *PdMATE16* has strong correlations with most of the flavones and chalcones, including kaempferol, apigenin, and lignans, while *PdMATE35* has strong correlations with 3–4 flavonol substances each, with *PdMATE35* strongly correlating with kaempferol. Therefore, we hypothesized that the *PdMATE16* and *PdMATE35* genes might be involved in the transport process of pigments in the yellow petals of *P. delavayi* var. *lutea* and play different transport functions.

### Molecular docking analysis of candidate MATEs

**Figure 4:**
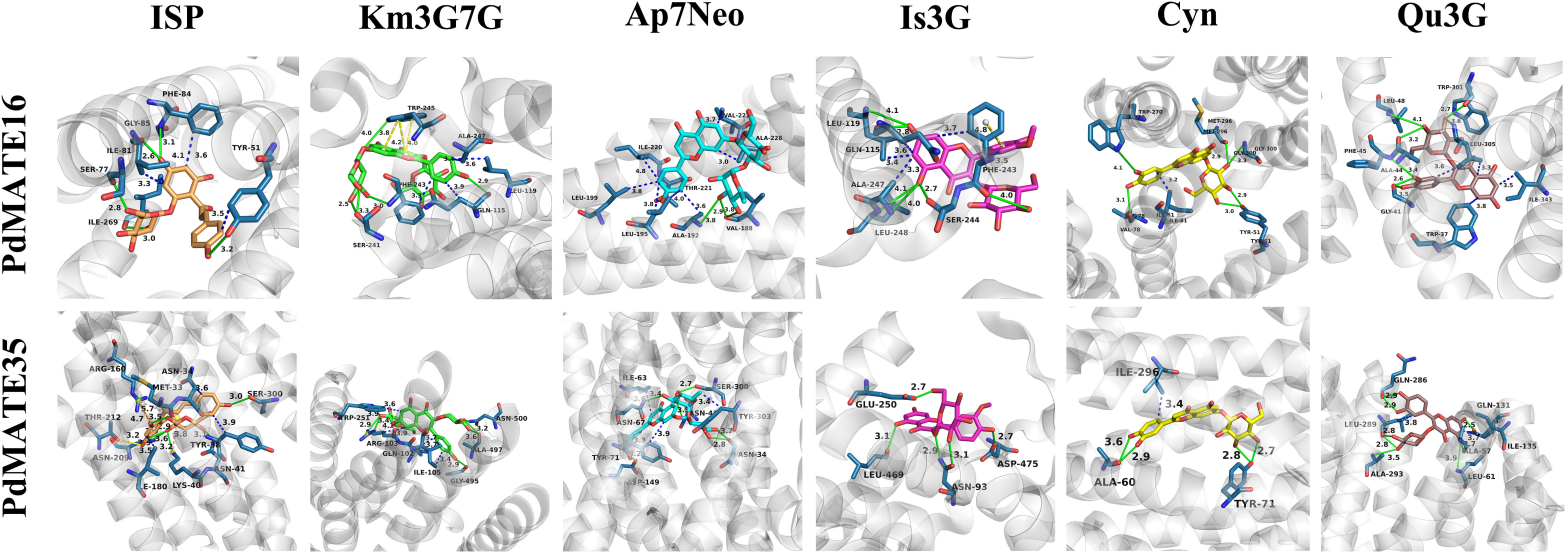
Molecular docking analysis of PdMATE16 and PdMATE35 with flavonoid compounds. Solid green lines show hydrogen-bonding interactions; salt-bridge interactions are shown by dashed yellow lines; π-Cation interactions are shown by dashed blue lines. ISP: isosalipurposide; Km3G7G: kaempferol 3,7-di-*O*-glucoside; Ap7Neo: apigenin 7-*O*-neohesperidoside; Is3G: isorhamnetin 3-*O*-glucoside; Cyn: cynaroside; Qu3G: quercetin 3-*O*-galloylglucoside.

In a previous study, we found that acylated flavonol glycosides, flavonoids, and chalcone glycosides (e.g., isosalipurposide) are the major color-presenting substances in *P. delavayi* var. *lutea* (Yuan et al., 2025). Therefore, we applied bioinformatics methods to modeling the structures of MATE16 and MATE35 and used molecular docking to calculate the binding free energies for the interaction of these transporter proteins with a variety of flavonoid substances (**Figure 4**). These substances include ISP, luteolin, apigenin, kaempferol, isorhamnetin, and quercetin. The data show that for all candidate transporter genes, all seven ligands have negative binding free energy (*ΔG*) values, indicating that they can bind these substrates (**Supplementary Table S2**). In particular, the ligands with the strongest binding ability for PdMATE16 and PdMATE35 were cymaroside (Cyn) and apigenin 7-*O*-neohesperidoside (Ap7Neo), respectively, with the second highest binding ability to ISP (isosalipurposide). This suggests that the two candidate MATEs have slightly different transporter preferences and may prioritize substrate translocation differently.

In addition, we visualized the molecular docking results and analyzed the key amino acid residues of PdMATE16 and PdMATE35 proteins interacting with the key color presenter ISP. These included TYR (51), SER (77A), ILE (81A), GLY (85A), and ILE (269) in PdMATE16, and MET (33), LYS (40), ASN (41), ASN (209), THR (212), and SER (300) in PdMATE35 (**Supplementary Table S3**). To further understand the functional roles of the two candidate MATE proteins, we mutated these sites and calculated the change in binding energy before and after mutation (**Supplementary Table S4**). The results showed that the binding affinity of all mutants was reduced compared to the wild-type protein. By analyzing the values of *ΔΔG*, we found that the PdMATE16-5 (ILE269Leu) mutant had the greatest effect on the free energy of ISP, Ap7Neo, and Isorhamnetin 3-*O*-glucoside (Is3G), a result that is consistent with the results of the previous gene and metabolite correlation analyses (**Figure 3**). In addition, the PdMATE35-2 (LYS40Ala) mutant had the greatest effect on the free energy of Ap7Neo, kaempferol 3,7-di-*O*-glucoside (Km3G7G), and Is3G. These results suggest that the ILE269 in PdMATE16, and LYS40 sites in PdMATE35 may play a key role in the transport of multiple flavonoid substances (**Supplementary Figure S6**).

### Optimal genetic transformation receptor selection

Since the genetic transformation system of *P. delavayi* var. *lutea* has not yet matured, gene function verification and other related experiments require heterologous expression with the help of a receptor with a well-established genetic transformation system. Model organisms such as Arabidopsis, tobacco, and maize are often used as genetic transformation receptors in experiments to explore the functional characteristics of genes. Therefore, we analyzed the genomic codons of different model organisms in comparison with the frequency of codon usage of the PdMATE gene. Codon preferences were considered to be more consistent between species when the ratio was between 0.5 and 2.0; otherwise, codon preferences were greater (**Supplementary Table S5**). The results showed that the number of codon usage frequencies of the PdMATE gene that differed significantly from those of the Arabidopsis, tobacco, and maize genomes was 16, 13, and 23, respectively, indicating that Arabidopsis and tobacco are more suitable for use as genetic transformation receptors for the PdMATE gene. Therefore, subsequently, we performed overexpression experiments of the candidate gene in tobacco to be used to verify the function of the gene.

### Cloning and sequence analysis of candidate MATE genes

*P. delavayi* var. *lutea* cDNA was used as a template, and full-length primers of *PdMATE16* and *PdMATE35* genes were specifically amplified to obtain sequences with lengths of 1461 bp and 1515 bp, respectively. After sequencing, the two genes had a complete ORF from start codon to stop codon, encoding 487 and 505 amino acids, respectively.

**Figure 5:**
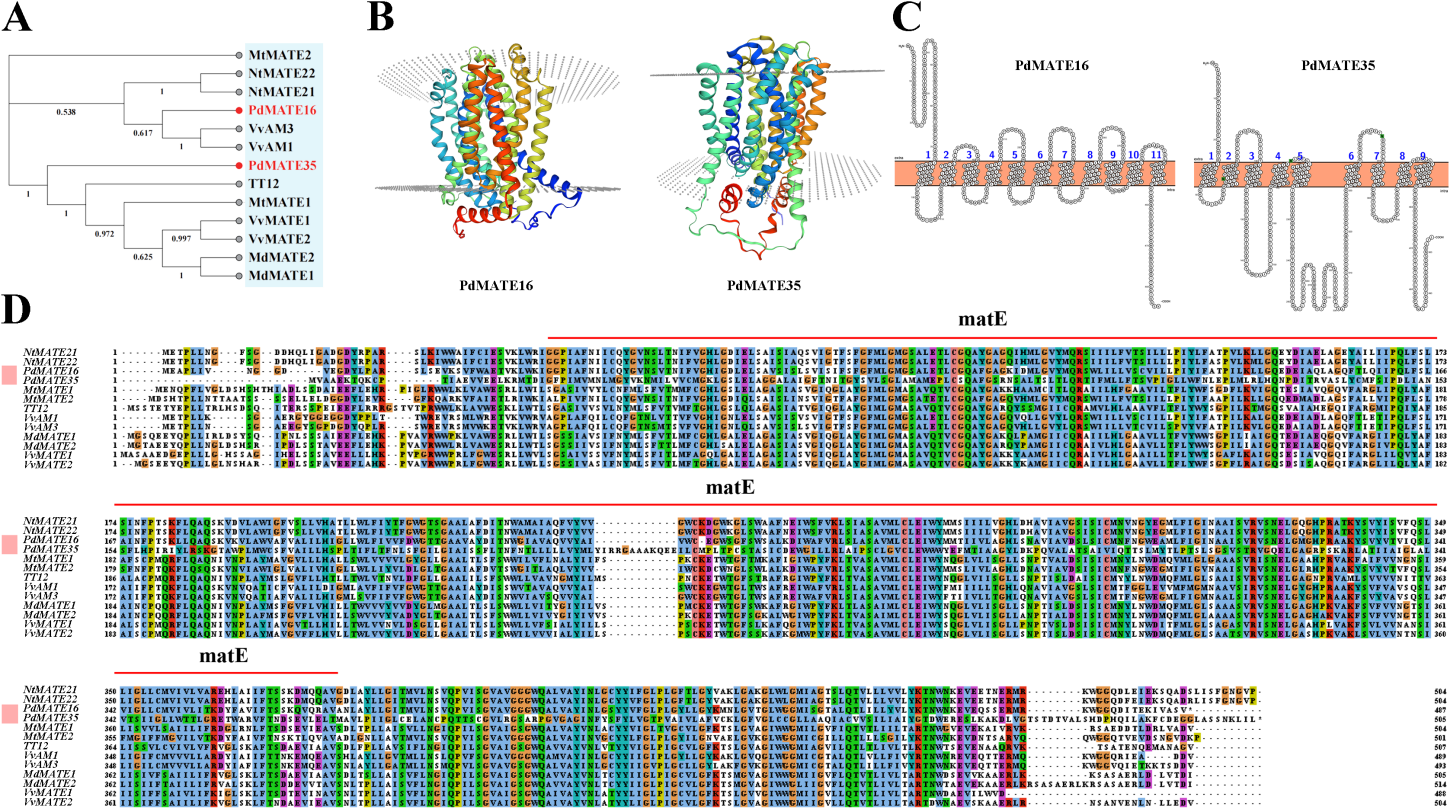
Cloning and sequence analysis of candidate PdMATE genes. **(A)** Phylogenetic tree of PdMATE16 and PdMATE35 with other identified flavonoid-related MATEs in plants was constructed using the neighbor-joining method. **(B)** Protein 3D structure of PdMATE16 and PdMATE35. **(C)** Predicted transmembrane helical structures of PdMATE16 and PdMATE35; 1–11 indicates the number of transmembrane helices of PdMATE. **(D)** Multiple comparisons of the amino acid sequence of PdMATEs with their homologs. Different colors represent different identities.

The conserved structural domains of the two MATE proteins were searched by Conserved Domain online software, and the results showed that both candidate MATE-encoded proteins have structural domains typical of the MATE superfamily (MatE), located between sequences 124–606 as well as sequences 778–1281 of the searched sequences, respectively (**Figure 5D**). The protein tertiary structure model was constructed using SWISS-MODEL, and the results showed that both PdMATE proteins contain a central cavity (**Figure 5B**). Meanwhile, in this study, the transmembrane region prediction of amino acids encoded by the PdMATE16 and PdMATE35 genes was performed by the online software Protter Webserver, and the results showed that most of the amino acid structures of the two proteins were transmembrane structures (**Figure 5C**). These central cavities and trans-helical structures ensure that the PdMATE proteins perform their transport functions stably.

Currently, a variety of MATE genes related to flavonoid transport have been validated in plants, such as *TT12, MtMATE1/2, VvAM1/3, VvMATE1/2, FFT, OsMATE1/2*, and *NtMATE21/22* (Marinova et al., 2007; Kryvoruchko et al., 2018; Pérez-Díaz et al., 2014; Tiwari et al., 2014; Gani et al., 2022). By constructing a phylogenetic tree, the results showed that PdMATE16 (MATE II) was first clustered in the same branch with genes transporter of flavonoids in grapes; PdMATE35 (MATE IV) was clustered with other sequences (**Figure 5A**). Multiple sequence comparison analysis showed that the two genes had high homology with genes with verified functions. SignalP 4.1 was applied to predict and analyze the signal peptides of the two candidate PdMATE family proteins (**Supplementary Figure S3**), and the results showed that the C and Y values of the PdMATE16 and PdMATE35 proteins were less than +0.5, with no significant change in the S value and no obvious signal peptide features, which indicated that both PdMATE family proteins did not contain signal peptides and belonged to the non-secretory proteins. Therefore, the two genes are strong candidates for participation in flavonoid transport in the petals of *P. delavayi* var. *lutea*.

### Heterologous overexpression of PdMATE16 or PdMATE35 promotes anthocyanin accumulation in tobacco

**Figure 6:**
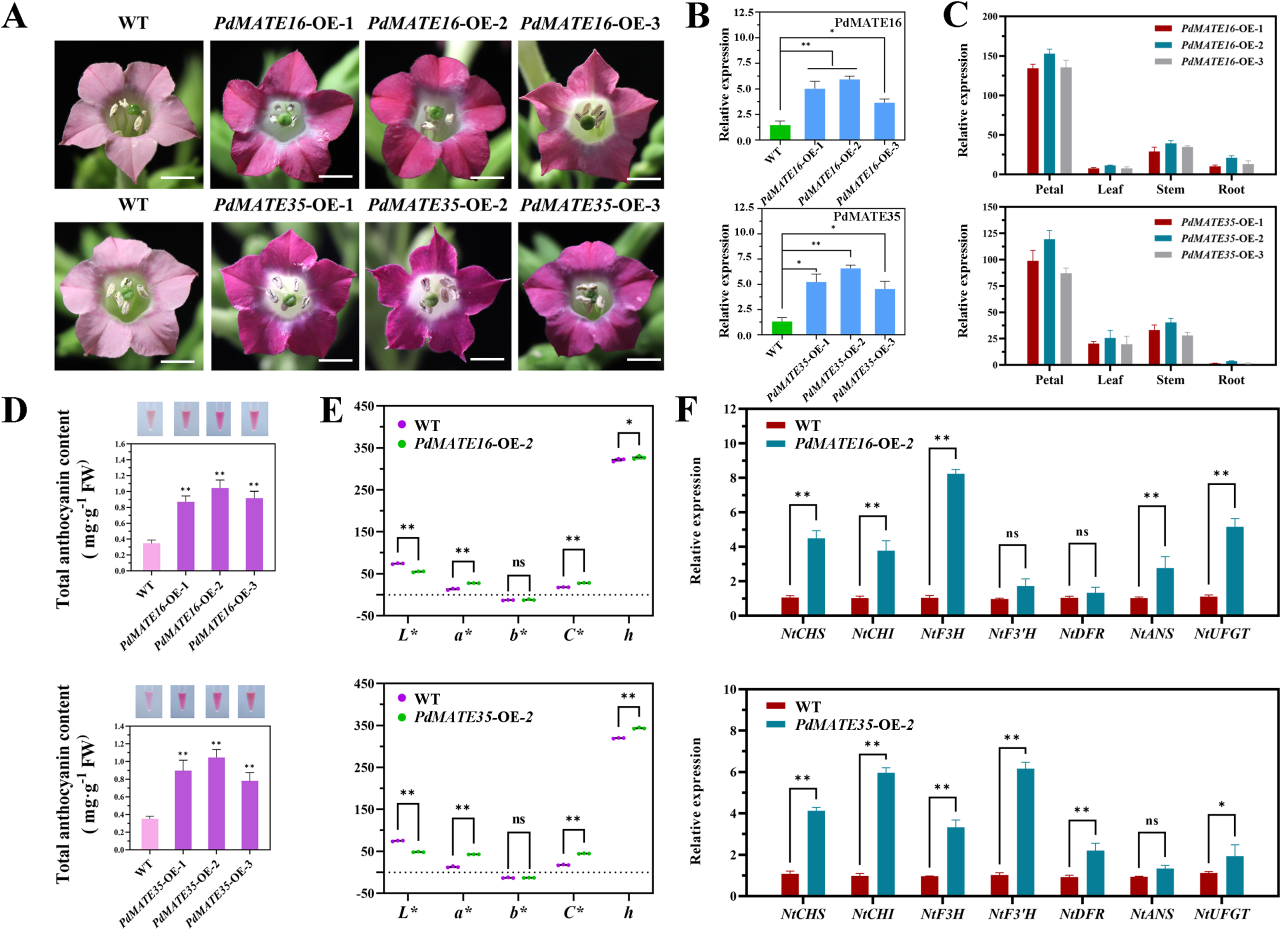
Effects of overexpression of *PdMATE16* and *PdMATE35* in tobacco. **(A)** Wild-type (WT) and overexpression of *PdMATE16* and *PdMATE35* in tobacco petals phenotypes. Bar = 2 cm. **(B)** The results of qRT-PCR detection in transgenic tobacco lines. **(C)** Temporal and spatial expression patterns of *PdMATE16* and *PdMATE35* in the three transgenic tobacco lines. **(D)** Total anthocyanin content was determined in petals of overexpression strains. **(E)** Over-represents the color index of the strain. L* denotes brightness. a* and b* denote red and yellow, respectively. C* denotes chromaticity. h denotes hue angle. **(F)** Expression patterns of endogenous tobacco genes in petals of WT and transgenic tobacco plants. Error lines indicate standard deviation in three biological replicates. Asterisks in **(B–D, F)** indicate significant differences. (Student’s t-test, *P < 0.05, **P < 0.01.).

To further validate the function of the candidate transporter proteins in plants, we performed overexpression experiments in the model plant tobacco. A total of six transgenic plants were obtained (**Figure 6A**). The phenotypic results showed that the flower color of transgenic tobacco was significantly deeper compared with WT. Among them, *PdMATE16* showed a deeper pink color than *PdMATE35*. Tissue-specific results showed that both *PdMATE16* and *PdMATE35* were significantly higher expressed in petals of transgenic lines than in stems, leaves and roots (**Figure 6C**). In order to further investigate the material basis of the color change, we determined the changes in the total anthocyanin content, and the results showed that the anthocyanin content in the petals of both *PdMATE16* and *PdMATE35* tobacco transgenic strains showed a significant increase, which was 2.51-3.02-fold higher than that of WT (**Figure 6D**). By measuring the expression of target genes in transgenic plants, we found that the expression of target genes was higher in *PdMATE16*-OE-2 and *PdMATE35*-OE-2 (**Figure 6B**). Therefore, we selected these two strains for further experimental analysis. The results of the floral color value assay showed significant changes compared with WT, except for the b* value, which was consistent with the phenotypic changes (**Figure 6E**). qRT-PCR results showed a significant 8.25-fold increase in *NtF3H* in *PdMATE16*-OE-2. The expression of other structural genes on the anthocyanin synthesis pathway was also up-regulated to varying degrees, e.g., *CHS, CHI*, and *UFGT* were up-regulated by 3.76 to 5.16-fold (**Figure 6F**). In *PdMATE35*-OE-2, *NtCHI* and *NtF3’H* were the most significantly up-regulated, at 5.96 to 6.16-fold of the content in the wild type.

### Silencing of PdMATE16 or PdMATE35 affects the content of multiple pigments in peonies

**Figure 7:**
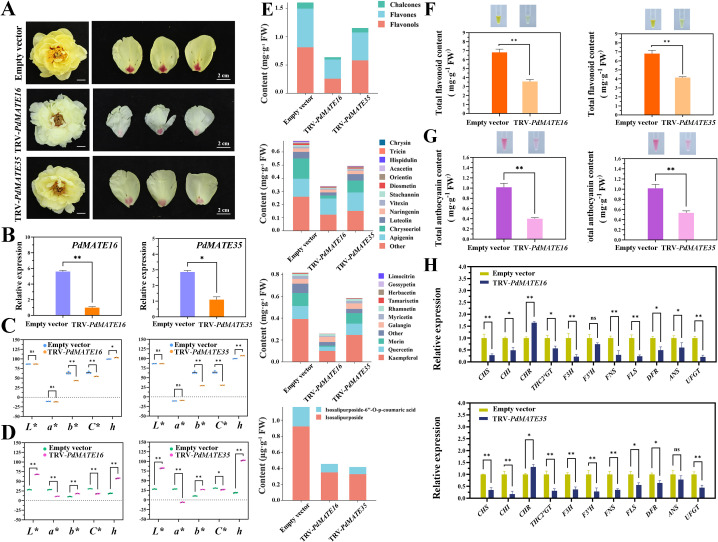
Effects of silencing of TRV empty vector, TRV-*PdMATE16* and TRV-*PdMATE35* in ‘High Noon.’ **(A)** Phenotypic observation of ‘High Noon’ peony petals after silencing TRV empty vector, TRV-*PdMATE16* and TRV-*PdMATE35*. Bar = 2 cm. **(B)** qRT-PCR detection results in silenced petals. **(C)** Color indices of petal regions after silencing TRV empty vector, TRV-*PdMATE16* and TRV-*PdMATE35*. L* denotes brightness. a* and b* denote red and yellow, respectively. C* denotes chromaticity. h denotes hue angle. **(D)** Color indices of the floral patch region after silencing TRV empty vector, TRV-*PdMATE16* and TRV-*PdMATE35*. **(E)** Trends of the contents of different flavonoid compounds in petals after silencing TRV empty vector, TRV-*PdMATE16* and TRV-*PdMATE35*. **(F)** Determination of total flavonoid content in petals after silencing TRV empty vector, TRV-*PdMATE16* and TRV-*PdMATE35*. **(G)** Determination of total anthocyanin content in the floral patch region after silencing TRV empty vector, TRV-*PdMATE16* and TRV-*PdMATE35*. **(H)** Expression patterns of structural genes and candidate transcription factors on the flavonoid synthesis pathway in petals after silencing TRV empty vector, TRV-*PdMATE16* and TRV-*PdMATE35*. Error lines indicate standard deviations in three biological replicates. Asterisks in **(A, C, D)** indicate significant differences. (Student’s t-test, *P < 0.05, **P < 0.01.).

In order to verify the transporter role of the candidate transporter genes in peony, we chose ‘High Noon’ variety, which is related to *P. delavayi* var. *lutea*, for the VIGS silencing experiment. The results showed that compared with the TRV empty vector group, the yellow color of the petals of peonies silenced with *PdMATE16* and *PdMATE35* was significantly reduced, and the spot areas also had different degrees of discoloration, among which the effect of *PdMATE16* in reducing the flower color was more obvious (**Figure 7A**). The expression of *PdMATE16* and *PdMATE35* in the silenced group was reduced to different degrees, by 82.3% and 61.7%, respectively, confirming that the silencing was effective (**Figure 7B**). The results of flower color value assay showed that in the petal parts of *PdMATE16* and *PdMATE35* silencing groups, the b* and C* values decreased and the h value increased, which proved that the yellow color was diminished and the saturation was reduced (**Figure 7C**); whereas in the floral spot parts, the L*, b*, and h values increased, and the a* and C* values decreased, which indicated that the saturation and the red color were diminished (**Figure 7D**), which was consistent with the results of the phenotypes that we observed.

By determining the total flavonoid content and total flavonoid fractions in the petal region, we found that: Not only the total flavonoid content was significantly reduced in the TRV -*PdMATE16* and *TRV-PdMATE35* treatment groups, but also the contents of various flavonoid substances were generally significantly reduced compared to the TRV empty vector group (**Supplementary Figure S7**). Among them, chalcone, flavone and flavonol contents were significantly reduced by 26.7-62.4%, 26.7-62.4% and 28.4-68.4% compared to the control group (**Figure 7E**). The levels of apigenin, chrysoeriol, luteolin, and naringenin in flavones and kaempferol, quercetin, morin, and galangin in flavonols, as well as both ISPs, were significantly reduced. In particular, silencing of *PdMATE16* resulted in a more significant decrease in the total flavonoid content in the petals, which was reduced by 47.7% compared to the TRV empty vector group (**Figure 7F**), a result that is consistent with the results of gene co-expression, which involves more genes for key flavonoid synthesizing enzymes in the module where *PdMATE16* is located. In addition, we performed pigmentation assays on the floral patch region (**Figure 7G**). The results of anthocyanin assay in the floral patch region showed that *PdMATE16* and *PdMATE35* were reduced by 60.7% and 47.7%, respectively, compared with the unloaded group. These results suggest that *PdMATE16* and *PdMATE35* gene silencing may inhibit the transport of multiple flavonoids in the petals.

The qRT-PCR results showed that after silencing the candidate genes, the structural genes related to flavonoid synthesis and transport in peony petals were decreased to different degrees (**Figure 7H**). In *PdMATE16* plants, the *CHS, F3H, FLS*, and *UFGT* genes were down-regulated to a more pronounced extent of 72.1%-79.3%. In PdMATE35, mainly upstream structural genes were more significantly down-regulated. For example, *CHI, THC2’GT* and *F3’H* were down-regulated by 83.5%, 69.2%, and 72.3%, in that order. Therefore, PdMATE16 and PdMATE35 may be involved in regulating the formation of flower color in *P. delavayi* var. *lutea* by regulating the transport of flavonoids.

**Figure 8:**
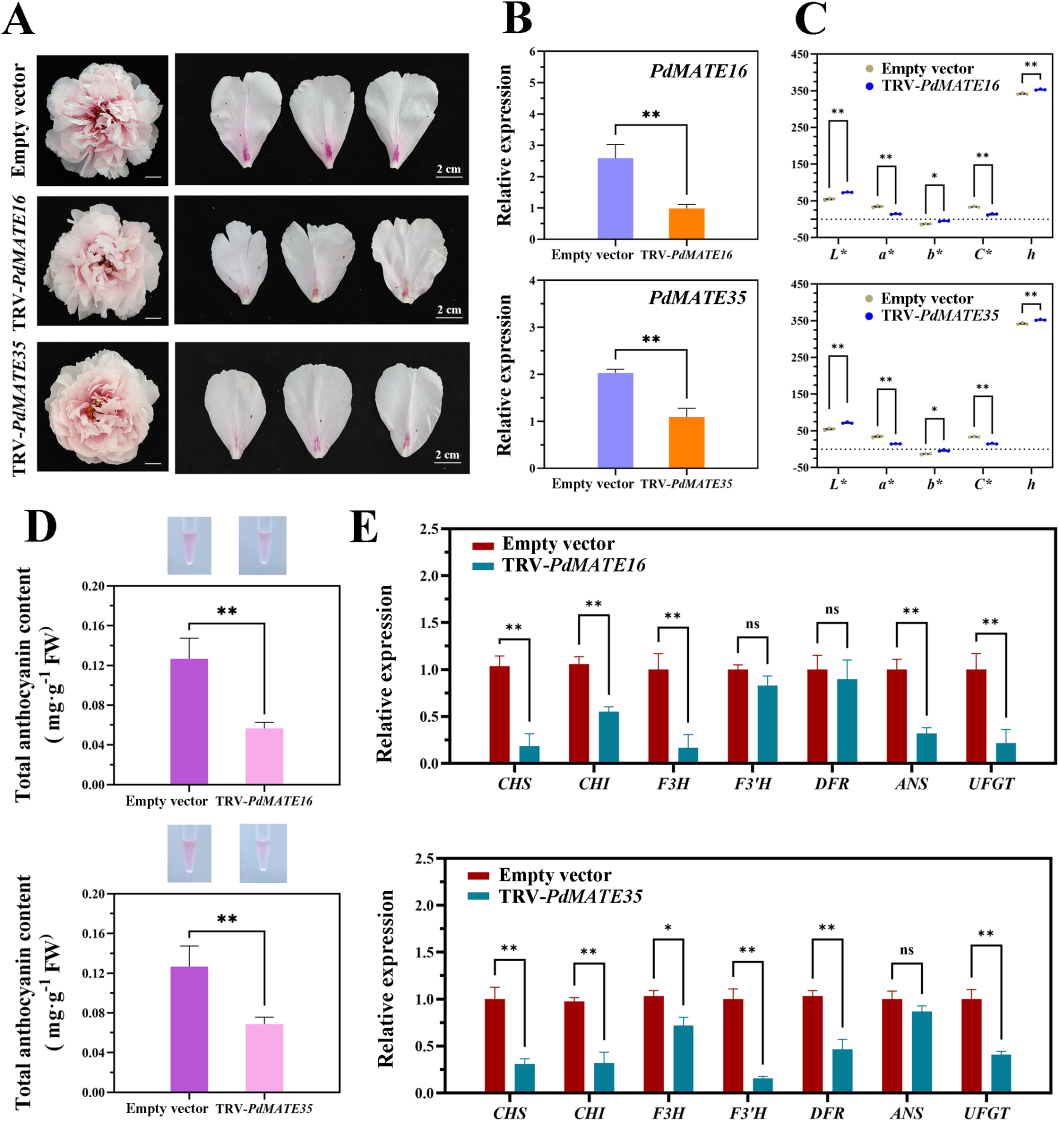
Effects of silencing of TRV empty vector, TRV-*PdMATE16* and TRV-*PdMATE35* in ‘Xue Ying Tao Hua.’ **(A)** Phenotypic observations of ‘Xue Ying Tao Hua’ peony petals after silencing TRV empty vector, TRV-*PdMATE16* and TRV-*PdMATE35*. Bar = 2 cm. **(B)** qRT-PCR -PCR detection results in silenced petals. **(C)** Color indices of the blotchy areas after silencing TRV empty vector, TRV-*PdMATE16* and TRV-*PdMATE35*. L* denotes luminance. a* and b* denote red and yellow, respectively. C* denotes chromaticity. h denotes the hue angle. **(D)** Determination of total anthocyanin content in the blotchy areas after silencing TRV empty vector, TRV-*PdMATE16* and TRV-*PdMATE35*. **(E)** Expression patterns of structural genes and transcription factors on the flavonoid synthesis pathway in petals silenced for TRV empty vector, TRV-*PdMATE16* and TRV-*PdMATE35*. Error lines indicate standard deviation in three biological replicates. Asterisks in **(B–E)** indicate significant differences. (Student’s t-test, *P < 0.05, **P < 0.01.).

Existing studies have shown that MATE has the function of transporter of anthocyanins; therefore, we conducted VIGS silencing experiments in the yellow-free peony variety ‘Xue Ying Tao Hua’. The results showed that the pink color faded significantly in peony petals silenced with *PdMATE16* and *PdMATE35* (**Figure 8A**). In the silencing group, not only the expression of the target genes was significantly reduced (**Figure 8B**), but also the red representation (a*) in the flower color value was significantly reduced (**Figure 8C**). In addition, the anthocyanin content at the petal base of the PdMATE16 and PdMATE35 silencing groups was reduced by 55.3% and 45.8%, respectively, which was consistent with the phenotype we observed (**Figure 8D**). Quantitative results showed that the expression of structural genes on the anthocyanin synthesis and transport pathways in peony petals was altered to different degrees (**Figure 8E**). Among them, the degree of down-regulation of upstream structural genes was more obvious in the *PdMATE16*-silenced group, with the most obvious decrease in the expression of *F3H*, which was 84.1%. the decrease of *F3’H* was obvious in the *PdMATE35*-silenced group, which was 83.9%. These results suggest that PdMATE16 and PdMATE35 also have the ability to transport anthocyanins.

## Discussion

MATE transporter proteins are widely present in a variety of organisms and are involved in the transport process of several secondary metabolites, including a variety of flavonoids (Marinova et al., 2007; Poustka et al., 2007). However, there are fewer relevant studies in ornamental plants, especially in peonies. Currently, little is known about how the PdMATE gene is involved in the transport process of flavonoid. In this study, we comprehensively analyzed the MATE gene family in *P. delavayi* var. *lutea*, functionally validated two of the MATE transporter proteins, and investigated the transporter roles they play in tobacco and peony petals.

### Transcriptome-wide characterization of the MATE gene family in *P. delavayi* var. *lutea*

Since *P. delavayi* var. *lutea* has not yet undergone genome sequencing, this study was conducted to identify the MATE gene family based on the transcriptome, and a total of 37 MATE genes were identified from *P. delavayi* var. *lutea*. The MATE gene family has been identified in several plants, including Arabidopsis, tobacco, wheat, potato, citrus, pear, cotton, radish, etc (Gani et al., 2021; Sheng et al., 2023; Huang et al., 2021; Zhang et al., 2021; Liu et al., 2022; Gao et al., 2024; Shen et al., 2024; M’mbone et al., 2018). The number of members of the MATE gene family ranges from 56 to 131. The low number of PdMATE genes identified in *P. delavayi* var. *lutea* may be due to the limited number of genes that can be identified in the transcriptome compared to the genome; in addition, there are some low-expressed genes that were eliminated. In addition, genome-wide duplications and tandem duplications also affect the number of gene families identified; for example, soybean has the highest number of MATE gene members due to the high rate of gene duplication in its archaeopolyploid genome (Liu et al., 2016). The MATE family is highly expanded in higher plants, reflecting the diversity of transport substrates for these proteins (Upadhyay et al., 2019).

The lengths of MATE proteins vary in different species. The Arabidopsis MATE protein contains 414–539 residues. The length of rice MATE proteins is 370–598 aa. In our study, the amino acid length of *P. delavayi* var. *lutea* proteins is 197–642 aa, suggesting that the MATE family in *P. delavayi* var. *lutea* may assume a more diverse range of functions. In addition, MATE usually contains 9–12 transmembrane helices, as well as two symmetrically distributed structural domains forming a delivery space (Kryvoruchko et al., 2018). The PdMATE proteins all have a helical structure, implying that these genes all have a delivery space and are capable of functioning as a transporter substrate (**Supplementary Figure S4**). An in-depth analysis of the PdMATE gene family revealed that the distribution of conserved motifs and gene structures showed significant heterogeneity across phylogenetic branches, whereas members within the same branch exhibited a high degree of homology (**Figure 2**). This finding is highly consistent with the results of MATE protein classification based on phylogenetic analysis, further validating the reliability of the classification.

The phylogenetic tree classified the PdMATE genes into four groups. Among them, the MATE II subfamily has the most members. Although the function of MATE in most *P. delavayi* var. *lutea* has not yet been determined, related studies in Arabidopsis have shown that MATE proteins are associated with the transport of flavonoids. Mutations in the FFT transporter protein lead to reduced levels of flavonol glucosides in Arabidopsis (Thompson et al., 2010). TT12, which also belongs to the MATE II subfamily, was the first protein identified in plants to be able to transport flavonoids and is localized to the vesicular membrane, which can be involved in the transport of flavan-3-ol glucosides in the Arabidopsis (Marinova et al., 2007). Multiple homologous proteins of TT12 share a similar function, and can be directly or indirectly involved in plant flavonoid transport. For example, heterologous overexpression of the *VvDTX41B* gene in grape in Arabidopsis *tt12* mutants restores proanthocyanidin deficiency (Huang et al., 2025). Strawberry FaTT12–1 functions as a transporter of phenolics. The above studies illustrate that the MATE proteins of this subfamily function to transport a wide range of flavonoids and have a degree of substrate specificity. Furthermore, although in the least abundant MATE IV subfamily, MATE45 in Arabidopsis is involved in the anthocyanin response to anthocyanin induction conditions (Kovinich et al., 2018). In summary, the PdMATE gene is widely associated with the pigment transport in the flower color of *P. delavayi* var. *lute*a, which can provide a molecular basis for breeding new yellow peony varieties.

### PdMATE16 and PdMATE35 are involved in the transport of multiple pigments in flower petals

Flavonoids, as a large group of important secondary metabolites in plants, play a role in various life activities and are involved in a variety of biological processes such as plant phenotypic coloration, reproductive growth, and stress resistance (Nabavi et al., 2018) The main color-presenting substances in *P. delavayi* var. *lutea* are flavones, flavonols and chalcones. Previous studies have shown that MATE transporter proteins can be involved in the transport of a variety of flavonoids, including anthocyanins and proanthocyanidins, and have also been shown to transport flavonols and flavones. For example, MtMATE1 and MtMATE2 in *Medicago truncatula* transport epicatechin 3’-*O*-glucoside into the vesicle (Zhao et al., 2011). In tobacco, down-regulation of *NtMATE21* and *NtMATE22* was reported to regulate flavonol content in transgenic tobacco (Gani et al., 2022). Therefore, we hypothesized that flavonoid-transporting MATE members are also present in *P. delavayi* var. *lutea*.

The MATE genes annotated by genomic data do not involve multiple periods of plant color presentation, and the genes screened by transcriptome may be more relevant. We screened two MATE transporter proteins from *P. delavayi* var. *lutea*, and molecular docking results showed that PdMATE16 and PdMATE35 were able to bind a variety of flavonoids, including substrates, ISP, luteolin, apigenin, kaempferol, isorhamnetin, and quercetin. After mutating ILE269 in PdMATE16, and LYS40 site in PdMATE35, respectively, the binding ability of MATE protein to various flavonoids was significantly reduced. The results of expression analysis in different tissues also showed that PdMATE16 and PdMATE35 were highly expressed in petals, and these results suggested that the two candidate genes might play a role in flavonoid accumulation.

The closer the kinship between different species, the more similar their codon preferences are, and vice versa, the more different they are (Liu et al., 2020). The analysis of codon preference can provide a reference for the matching of exogenous genes and receptors in heterologous expression, which can enhance the success rate of heterologous expression to a greater extent (Li et al., 2023). By analyzing the codon preference pattern of *P. delavayi* var. *lutea* PdMATE, Arabidopsis and tobacco are more suitable to be the genetic transformation receptors of the PdMATE gene. Therefore, we performed heterologous overexpression in tobacco, and the results showed that the content of anthocyanins in petals of *PdMATE16* and *PdMATE35* transgenic lines was significantly increased. In contrast, silencing of *PdMATE16* and *PdMATE35* in ‘High Noon’, a peony variety close to *P. delavayi* var. *lutea*, resulted in the yellow color of the petals and the purple color of the spots being diminished, and reduced the content of anthocyanins and total flavonoids in different regions. Silencing in the peony variety ‘Xue Ying Tao Hua’, which does not contain yellow color, only affected the anthocyanin content, which was significantly reduced. Several studies have confirmed that overexpression of transporter proteins in plants affects the content of secondary metabolites. Transient overexpression of *PbrMATE9* in pear promoted the accumulation of anthocyanin glycosides in the pericarp (Sheng et al., 2023). In tobacco, lines overexpressing *NtMATE21* and *NtMATE22* accumulated significantly higher flavonol content (kaempferol 3-*O*-rutinoside, rutin, kaempferol, and quercetin) compared to empty vector and WT control plants (Gani et al., 2022). In grape, overexpression of *VvGSTU60* resulted in a significant increase in proanthocyanidin content (Huang et al., 2025). These results are consistent with our findings and further indicate that PdMATE16 and PdMATE35 are involved in the accumulation of flavonoids in peonies and are capable of transporting anthocyanins and other flavonoids. In addition, these transport substrates all have pharmacological activity, and this result also provides new possibilities for breeding peonies that are both ornamental and medicinal.

At present, the flavonoid synthesis pathway in peony has been studied more thoroughly, and the roles of various structural genes have been verified (Luo et al., 2021; Shi et al., 2022; Luo et al., 2022; Qi et al., 2020; Gu et al., 2019). However, its downstream transport mechanism still remains to be investigated, especially in the yellow lineage varieties. In this study, predictions showed that both PdMATE16 and PdMATE35 were localized to the plasma membrane, suggesting that these transporter proteins may be involved in the transport of flavonoids between cells, supporting the speculation that they play a transporter function. Thus, genetic manipulation of transporter proteins could regulate the cellular content of flavonoids, which in turn may affect flavonoid-mediated signaling. It has been shown that MATE can affect the expression levels of genes related to structures on the flavonoid pathway. Silencing of *PhMATE1* in *Petunia tabacum* down-regulates the expression of structural genes of the anthocyanin synthesis pathway, including *PhF3’5’ H, PhDFR*, and *PhUFGT* (Yuan et al., 2023). Similarly, knockdown of *NtMATE* in tobacco resulted in down-regulation of the expression of related structural genes, including *NtCHI1, NtCHI2, NtF3H1, NtF3H2, NtFLS1*, and *NtFLS2* (Gani et al., 2022). In this study, silencing of *PdMATE16* and *PdMATE35* in ‘High Noon.’ peony also significantly down-regulated the expression of structural genes on the flavonoid synthesis pathway, including *CHS, F3H, FLS*, and *UFGT*, as well as *CHI, THC2’GT*, and *F3’H*. These results suggest that when flavonoids already synthesized in plants are blocked in translocation, there may be feedback mechanisms that can maintain intracellular material homeostasis by regulating the expression of structural genes downstream of the synthesis pathway (Tiwari et al., 2014). In addition, in this study, the expression of structural genes in the anthocyanin synthesis pathway (*NtF3H, NtCHI*, and *NtF3’H*) was significantly up-regulated after overexpression of *PdMATE16* and *PdMATE35* in tobacco to promote the anthocyanin synthesis pathway, which we hypothesized could be due to the fact that the substrate was transporter ized by the excess amount of substrate, and then reversedly promoted the cytoplasm for more anthocyanin synthesis.

### Prediction of potential regulatory networks for PdMATE16 and PdMATE35

Transcription factors play a central role in the regulatory mechanism of gene expression by specifically binding to cis-acting elements in the promoter region to achieve transcriptional regulation of target genes. Studies have shown that these regulatory proteins have multiple biological functions in plant life, not only in the fine regulation of growth and development, but also in plant coloration and secondary metabolite transport (Wu et al., 2024). Recent advances have revealed that the expression pattern of the MATE gene family is regulated at multiple levels by transcription factors, a phenomenon that has been demonstrated in a variety of plant species. Under Fe deficiency stress, MdMYB58 and MdSAT1 are involved in the plant response by regulating the expression of *MdMATE43* in apple (*Malus pumila*) (Wang et al., 2018). The MYB genes *TT2a, TT2b*, and *MYB14* and the bHLH gene *TT8* in lotus are significantly associated with the expression of the proanthocyanidin transporter gene *MATE1*, which may potentially regulate the transport of flavonoids in the petals (Escaray et al., 2017). In Japanese apricot (*Prunus mume*), the expression of *GST1, MATE, NAC*, and the MBW complex was significantly higher in the red variety than in the green variety, and it is hypothesized that these encoded genes may be collectively involved in anthocyanin transport (Ni et al., 2018). However, relevant studies involving the MATE transporter process in peony have not been carried out.

Using WGCNA analysis, the identified genes can be tightly linked to a certain function, and the method is currently widely used to predict the potential regulatory mechanisms existing between genes (Xie et al., 2019). In this study, a total of multiple transcription factors, including 14 MYB,7 BHLH and 2 WRKY, were screened as candidate genes to regulate *PdMATE16* and *PdMATE35*. Three PdMYBs (Isoform0018896; Isoform0022146; Isoform0033519) were found to have high homology with MYB transcription factors in other species by constructing evolutionary trees, suggesting that they may have similar roles. Among them, *MdMYB1*, which regulates structural genes for anthocyanin biosynthesis in red apple, is also able to activate MdMATE-LIKE1 as well as the promoters of *MdGSTF6* transporter genes (Jiang et al., 2019; Hu et al., 2016). AtPAP1 can be involved in flavonoid transporter by regulating the expression of the *AtMATE* gene. Overexpression of MYB transcription factors involved in proanthocyanidin biosynthesis, i.e., VvMYBPA1 and VvMYBPA2, enhances the expression of the MATE transporter protein VvTT12 in grapes (Bogs et al., 2007; Terrier et al., 2009). In addition, transcriptome analysis also revealed that all three PdMYB transcription factors were significantly highly expressed during the S2, which was positively correlated with the accumulation of flavonoids and the expression pattern of *PdMATE35*, so it is reasonable to speculate that they may regulate the expression of MATE during petal coloration, thus affecting the flower color of peony. However, the specific regulatory mechanism is still unclear and further relevant studies are needed. The potential transcription factors predicted by WGCNA may provide a molecular basis for further exploration of the involvement of PdMATE genes in the mechanism of floral pigment transport.

## Conclusion

A total of 37 MATE proteins were identified from the transcriptome of *P. delavayi* var. *lutea* in this study, which were classified into four subfamilies. Based on phylogenetic relationships and molecular docking, we screened two proteins, PdMATE16 and PdMATE35, which might be involved in flavonoid transport. Tobacco overexpression experiments and peony VIGS silencing experiments demonstrated that PdMATE16 and PdMATE35 could participate in the transport of a variety of flavonoid compounds such as flavones, flavonols, chalcones, and anthocyanins in petals. In addition, co-expression network analysis revealed potential regulatory relationships between the two candidate MATE proteins and some transcription factors (MYB, BHLH and WRKY). These results provide a reference for further research on the function of MATE transporters and the regulatory network of peony pigment synthesis and accumulation. At the same time, they support further breeding work to develop peony varieties that are both ornamental and pharmacologically active.

## Data Availability

Publicly available datasets were analyzed in this study. This data can be found here: PRJNA1240698.
