# Supplementary material for: PdMATE16 and PdMATE35 are essential for the accumulation of multiple flavonoids in tree peonies
Source: Front Plant Sci. 2025 Nov 19;16:1703357. doi: 10.3389/fpls.2025.1703357 (PMC12672446; doi:10.3389/fpls.2025.1703357)
Supplement: Supplementary file 1 [file DataSheet1.docx]

**
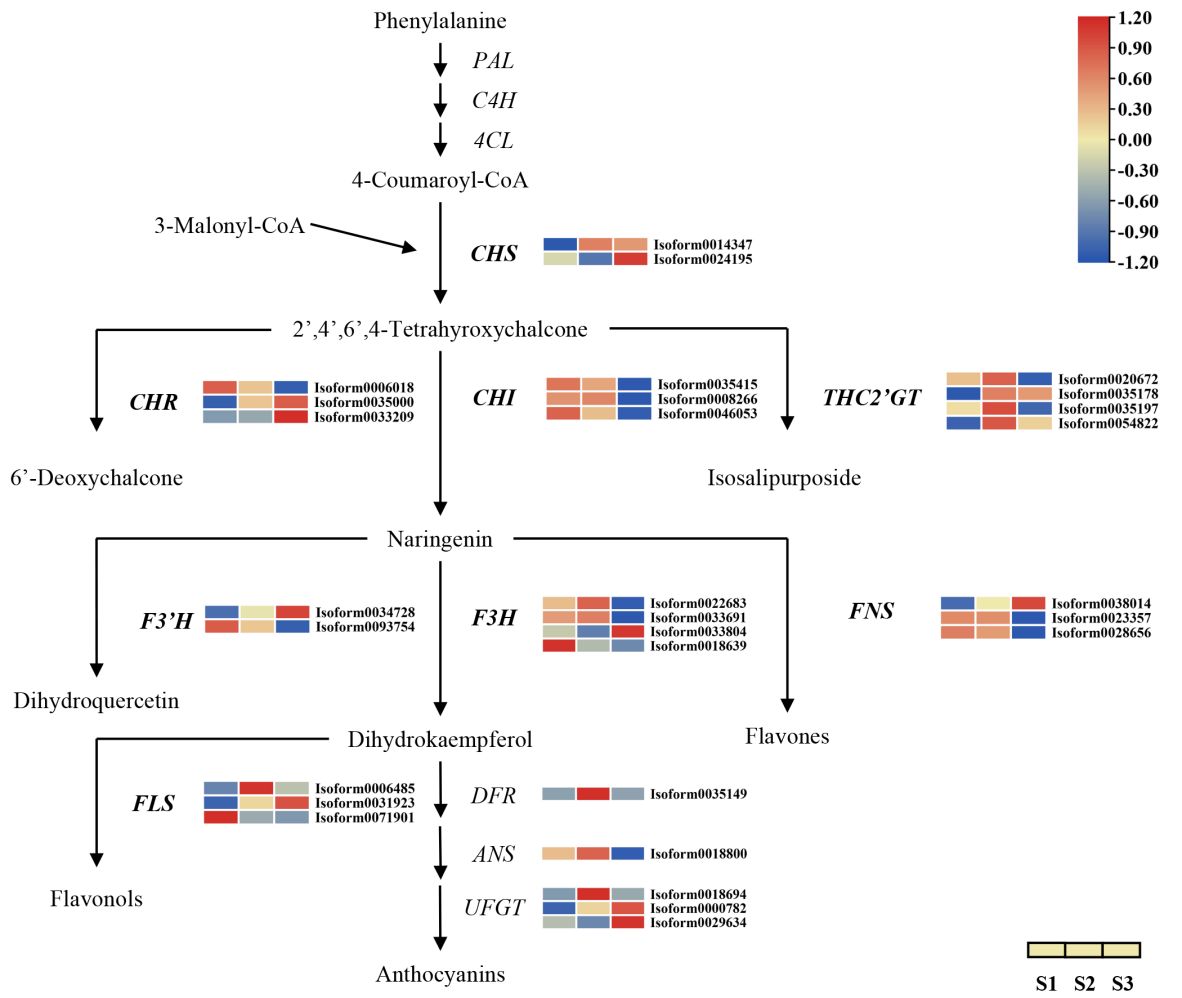
**

**Fig. S1.** **Expression heatmap of DETs for flavonoid biosynthesis in *P. delavayi* var. *lutea* during yellow pigment development.** The mean FPKM values for the structural s were calculated from three biological replicates for each sampling point (S1, S2, and S3). The progression of the colour scale from blue to red represents an increase in the FPKM values. *CHS*: chalcone synthase; *CHI*: chalcone isomerase; *CHR*: chalcone reductase; *THC2’GT*: chalcone 2´-glucosyltransferases; *F3H*: flavanone 3-hydroxylase; *F3’H*: flavonoid 3´-hydroxylase; *FNS*:flavone synthase; *FLS*: flavonol synthase; *DFR*: dihydroflavonol 4-reductase; *ANS*: anthocyanidin synthase; *UFGT*: flavonoid 3-*O*-glucosyltransferase.

**
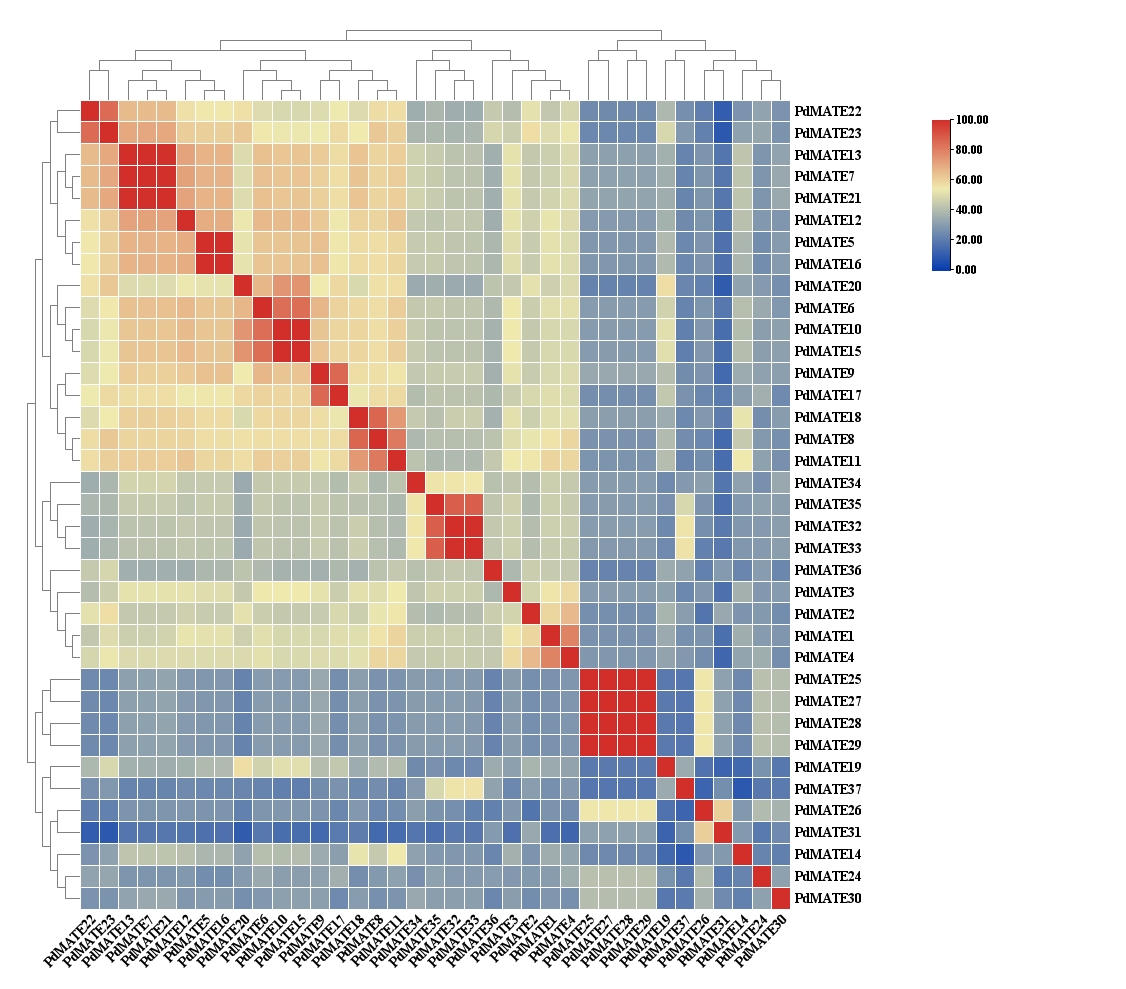
**

**Fig. S2. Similarity alignment between PdMATE proteins.**

**
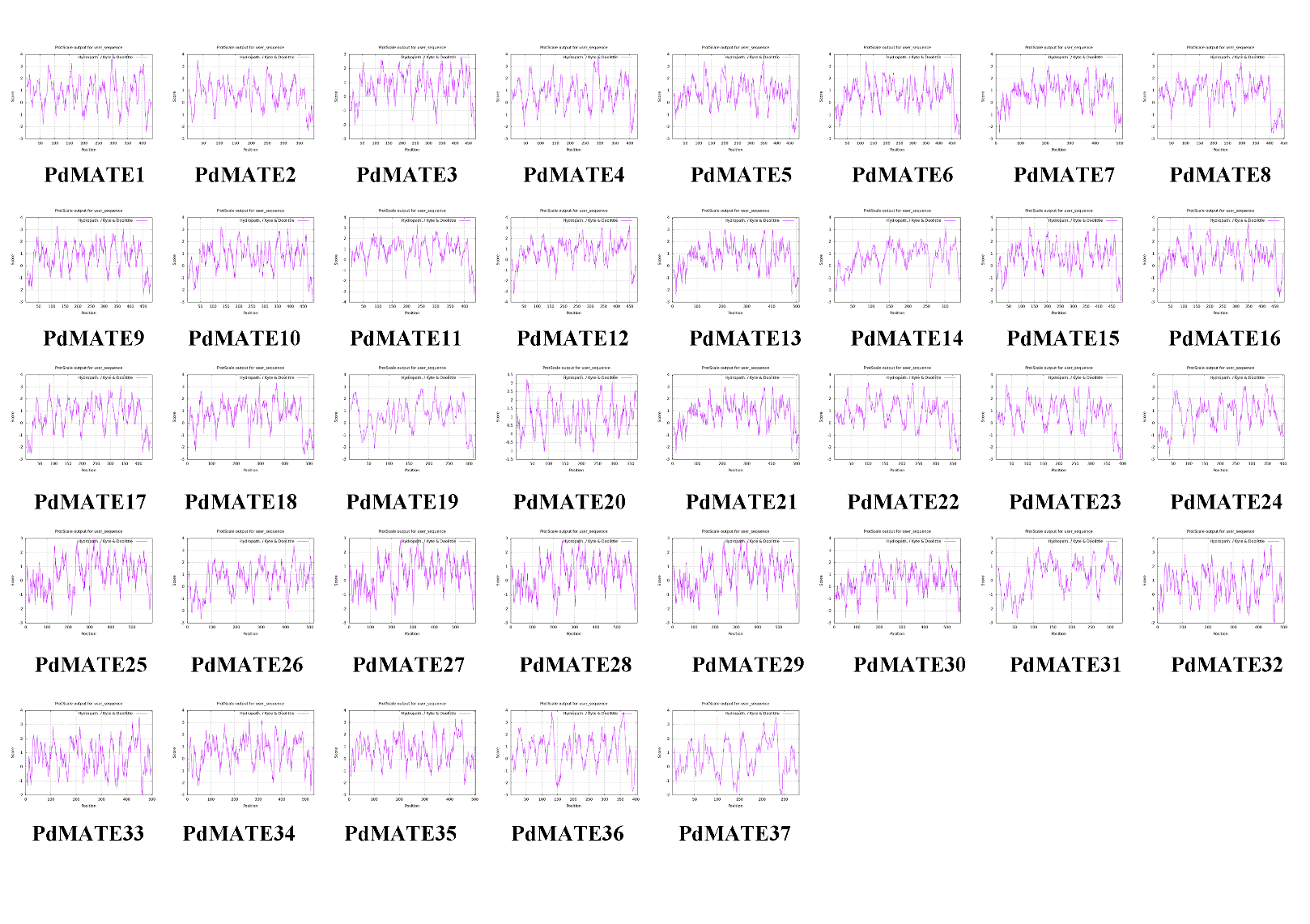
**

**Fig. S3. Analysis of Hydrophobicity of PdMATE Family Proteins.**

**
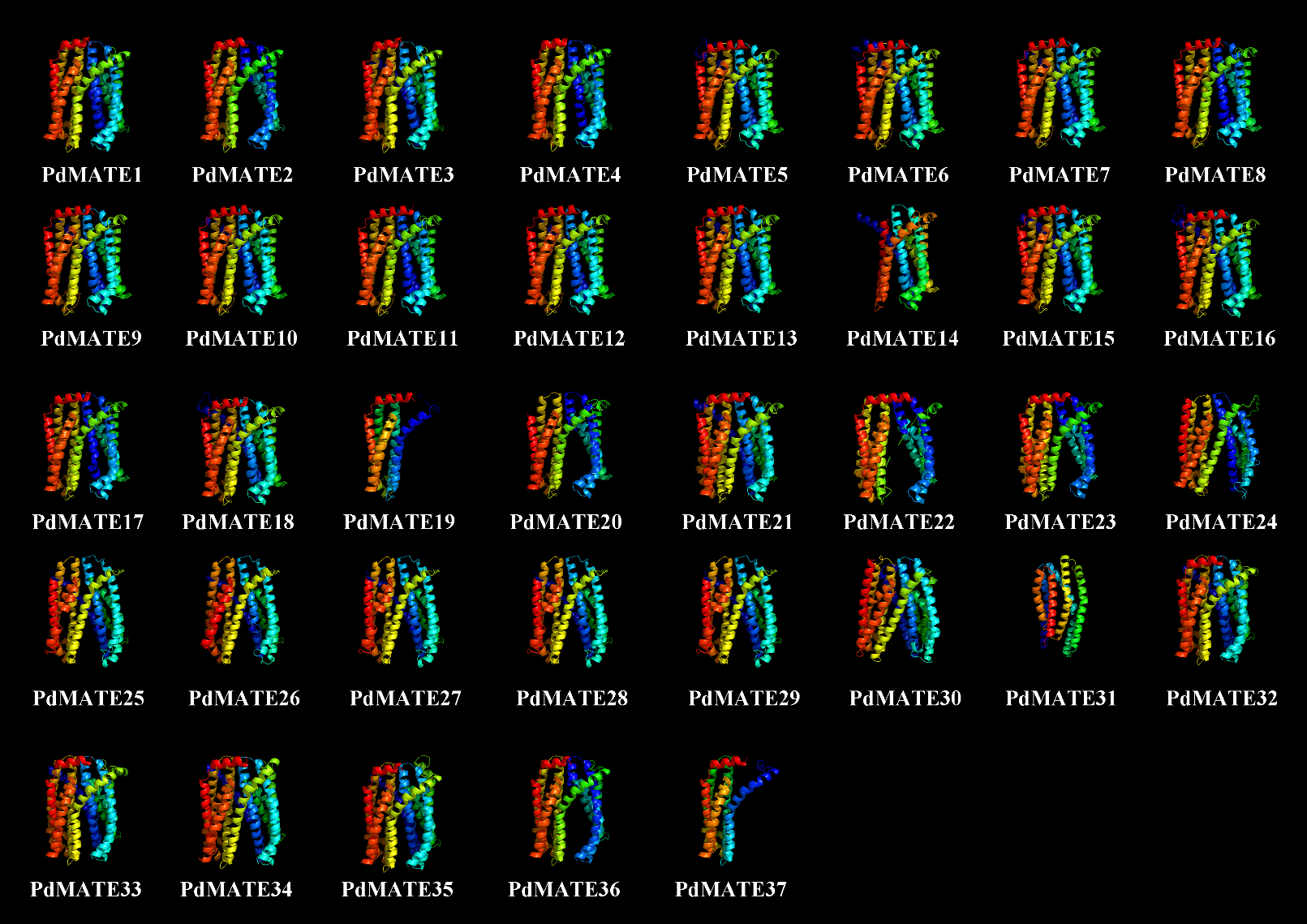
**

**Fig. S4. Predicted protein structures of the PdMATE gene family.**


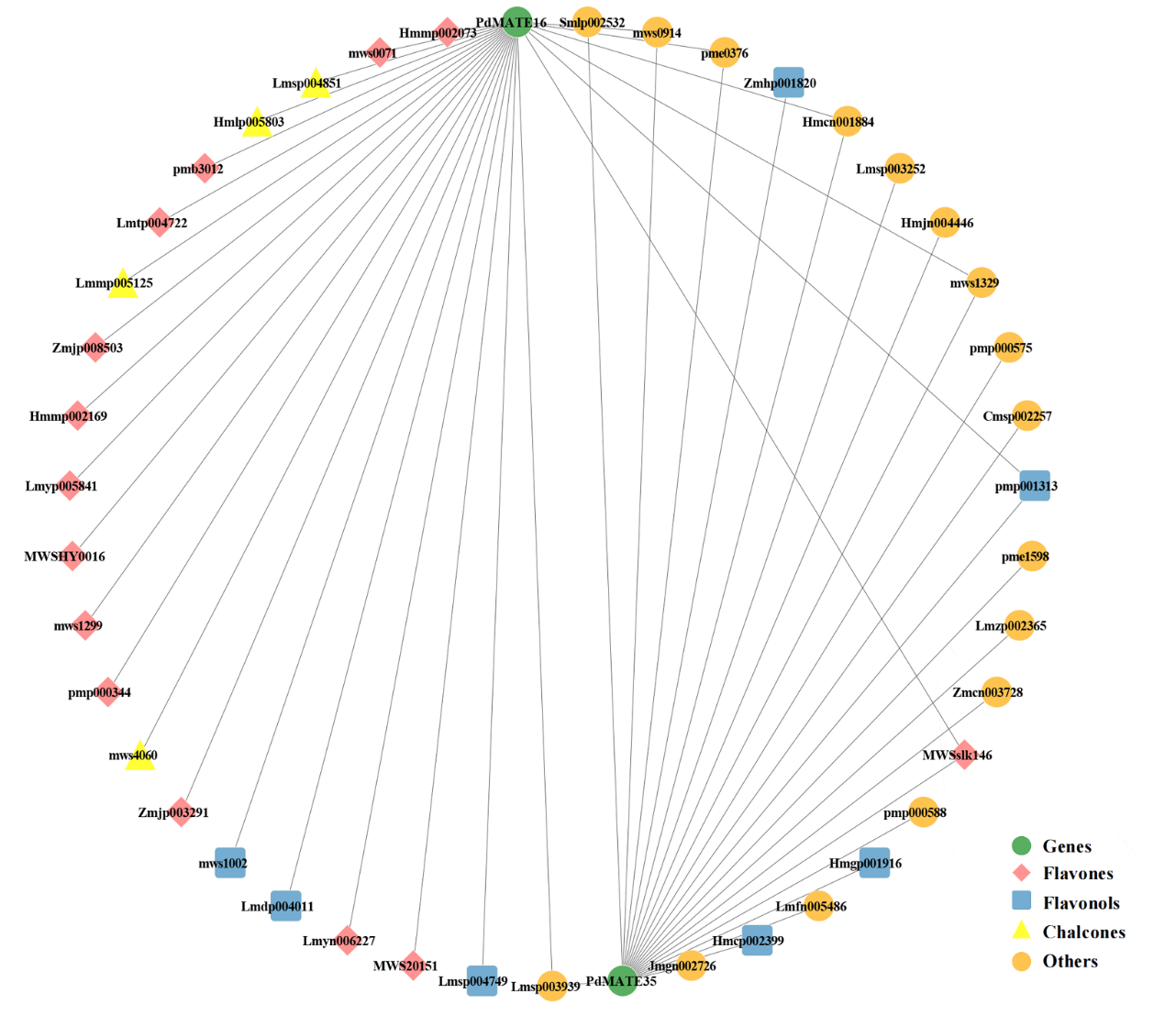


**Fig. S5. Correlation analysis of PdMATE genes with key metabolites.** Different colors represent different classes of flavonoids.


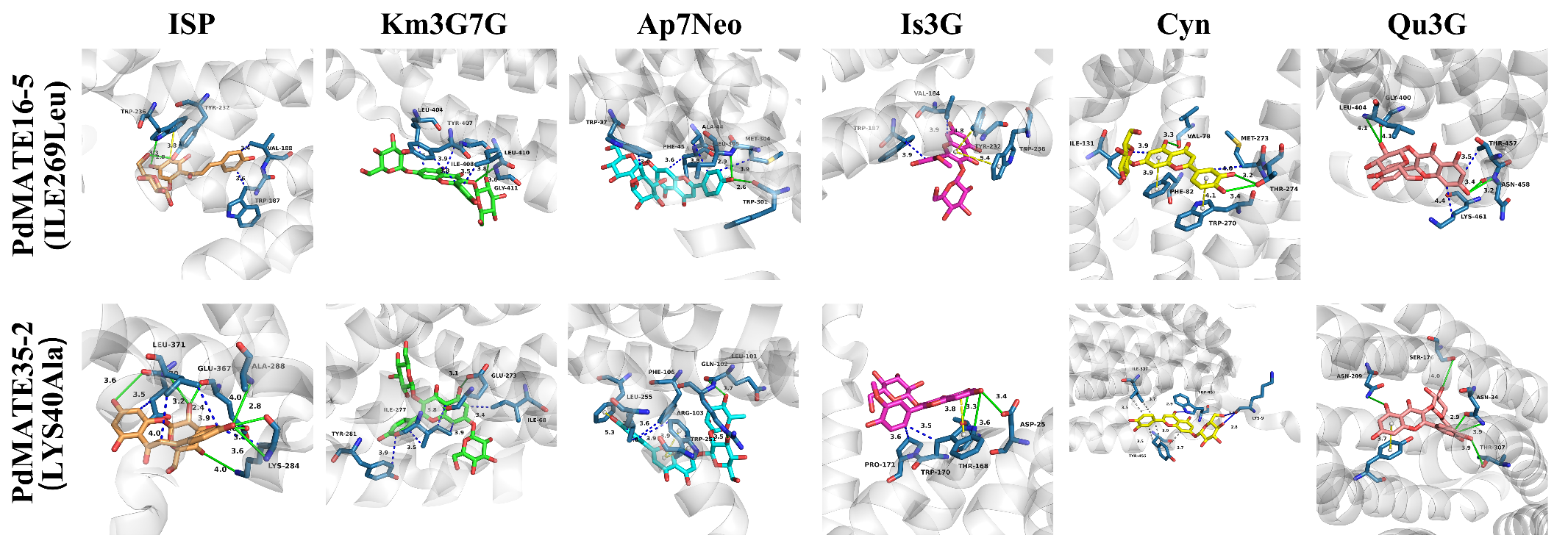


**Fig. S6. Molecular docking analysis of PdMATE16-5 (ILE269Leu) and PdMATE35-2 (LYS40Ala) with flavonoid compounds.** Solid green lines show hydrogen-bonding interactions; salt-bridge interactions are shown by dashed yellow lines; π-Cation interactions are shown by dashed blue lines. ISP: isosalipurposide; Km3G7G: kaempferol 3,7-di-*O*-glucoside; Ap7Neo: apigenin 7-*O*-neohesperidoside; Is3G: isorhamnetin 3-*O*-glucoside; Cyn: cynaroside; Qu3G: quercetin 3-*O*-galloylglucoside.

**
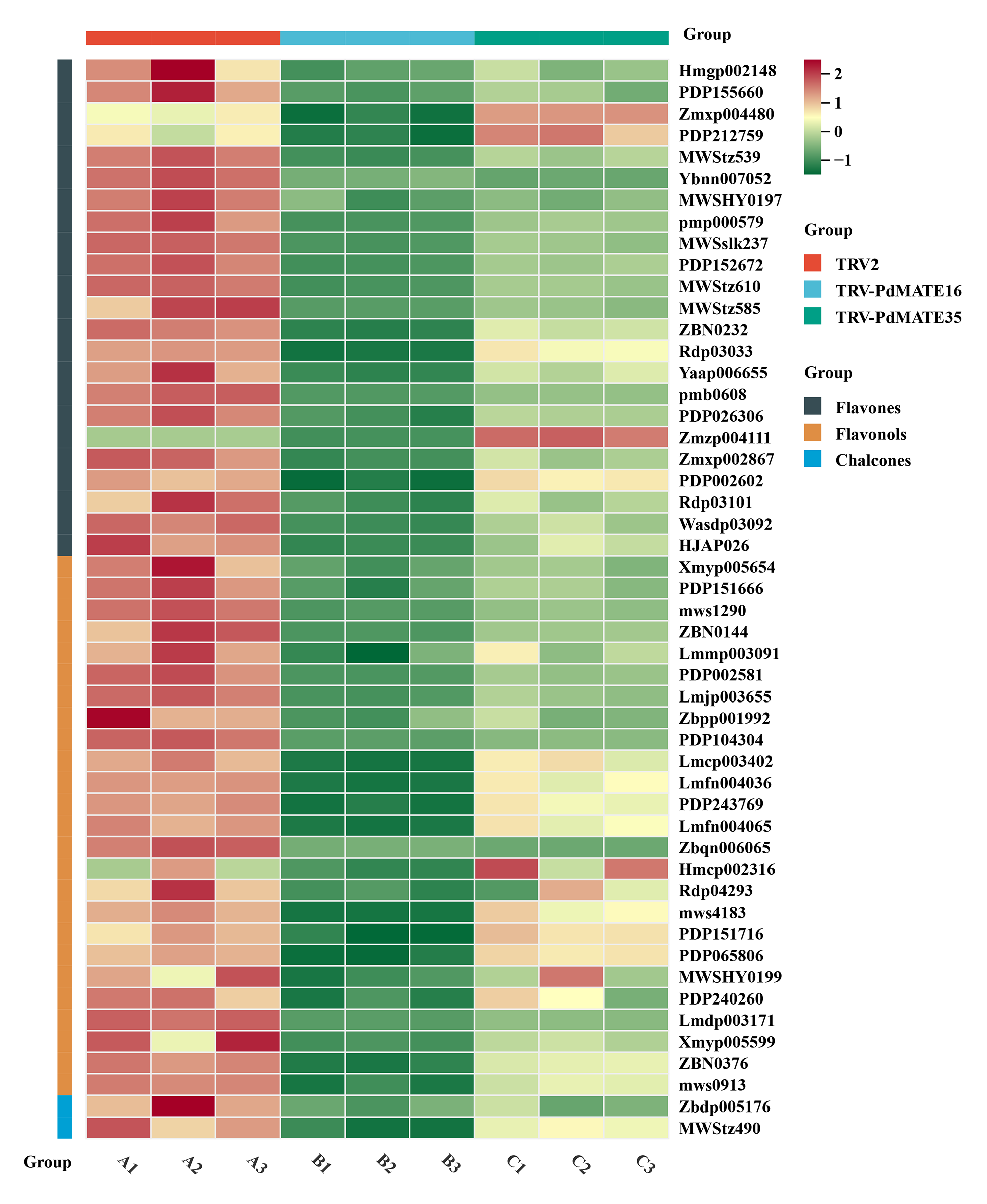
**

**Fig. S7. Thermogram analysis of each flavonoid fraction in petals after silencing TRV empty vector, TRV-*PdMATE16* and TRV-*PdMATE35*.**
